# Supplementary material for: Formation of H3+ from ethane dication induced by electron impact
Source: Commun Chem. 2020 Nov 9;3:160. doi: 10.1038/s42004-020-00415-9 (PMC9814254; doi:10.1038/s42004-020-00415-9)
Supplement: Supplementary file 1 — Supplementary Information [file 42004_2020_415_MOESM1_ESM.pdf]

Supplementary information

Formation of H_3^+ from Ethane Dication Induced by Electron Impact

Yu Zhang, Baihui Ren, Chuan-Lu Yang, Long Wei, Bo Wang, Jie Han, Wandong Yu, Yue Ying Qi,

Yaming Zou, Li Chen, Enliang Wang, and Baoren Wei

Supplementary Note 1. Coincidence TOF map and ion yields

Coincidence channels are identified through the ion-ion coincidence time-of-flight (TOF) map, i.e. a density plot of the TOF of the first ion *vs* that of the second one. Supplementary Figure 1 shows the full coincidence TOF map for dissociation of ethane dications. In addition to the interested channels $H_n^+ + C_2H_{6-n}^+$ (n=1-3), there are many other fragmentation channels involving C-H or C-C bond breakages. The counts of all of these channels are estimated in Supplementary Table 1. For the C-C bond-breakage channels, the coincidence stripes are partly overlapped, so particular care should be taken when counting the number. Following Supplementary Table 1, branching ratios among the H_n^+ ions and two-body H_n^+ channels produced by the dissociation of $C_2H_6^{2+}$ dications are estimated in Supplementary Table 2.

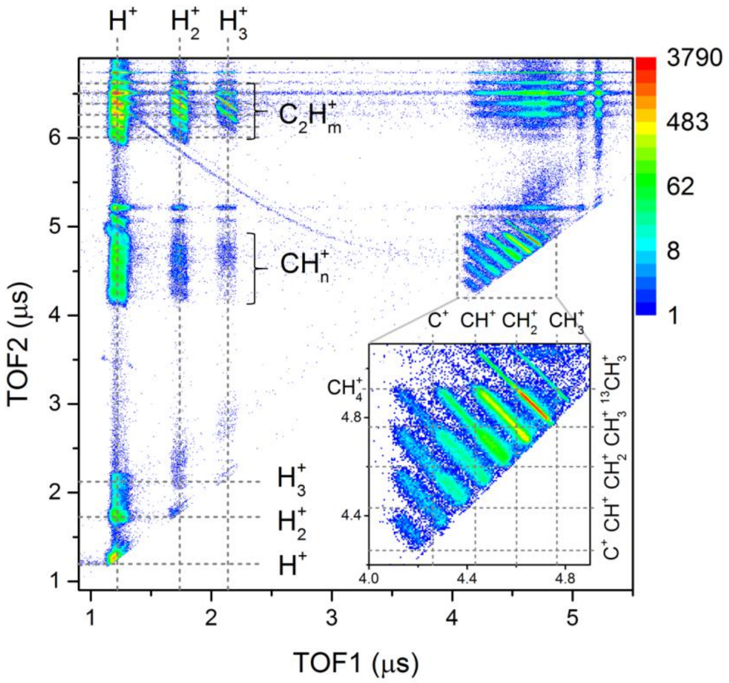

Supplementary Figure 1. Full ion-ion coincidence TOF map for the dissociation of $C_2H_6^{2+}$ dications induced by 300 eV electron impact. The inset is an enlarged spectrum for ion-pairs where the C-C bond of $C_2H_6^{2+}$ was broken.

Supplementary Table 1. Number of counts of ion-pairs formed by the dissociation of $C_2H_6^{2+}$ dications.

1st ion / 2nd ion	H^+	H_2^+	H_3^+	1st ion / 2nd ion	C^+	CH^+	CH_2^+	CH_3^+
$C_2H_5^+$	711	/	/	$^{13}CH_3^+$	/	/	/	2496
$C_2H_4^+$	14121	16743	/	CH_4^+	/	/	7460	/
$C_2H_3^+$	98814	48834	46943	CH_3^+	1835	11500	55866	118828
$C_2H_2^+$	86439	27154	10656	CH_2^+	3763	7576	11294	/
C_2H^+	45825	3986	352	CH^+	2664	2928	/	/
C_2^+	20843	1049	29	C^+	839	/	/	/
CH_n^+	67853	2727	248					
H_3^+	2314	81	22					
H_2^+	16088	433	/					
H^+	76070	/	/					

Supplementary Table 2. Branching ratios among the H_n^+ ions and two-body H_n^+ channels produced by the dissociation of $C_2H_6^{2+}$ dications in the present and previous studies.

H_n^+ ion	Present	Intense laser Ref. [1]	200 eV e^- Ref. [2]	580 keV C^+ Ref. [3]	200 eV e^- + CH_4 Ref. [2]
H^+	76.0	65/64	82.3	~79	96.31
H_2^+	15.3	20/15	11.6	~14	3.67
H_3^+	8.7	15/21	6.1	~7	0.02
Two-body H_n^+ channel	Present	Intense laser Ref. [4]	200 eV e^- Ref. [2]	300 eV e^- + C_2H_4 Ref. [5]	300 eV e^- + CH_4 Ref. [5]
$H^+ + C_2H_5^+$	1.1	~5	0	78.30	79.07
$H_2^+ + C_2H_4^+$	26.0	~7	26.2	21.21	20.73
$H_3^+ + C_2H_3^+$	72.9	~88	73.8	0.49	0.20

Supplementary Note 2. Theoretical channel yields

Note that in the present study we just simulated the dissociation dynamics of ethane dication in the ground state, which has been proven crucial to the formation of H_3^+ ions. A total of 1000 trajectories was computed and 230 out of them showed H_3^+ formation. The simulation results at 500 fs showed that H_2 is produced with fractional charge (mostly 0.5e). Thus the channels ending up with H_2 (might in neutral and charge states) correspond to H_2^+ + $C_2H_4^+$ and H_2 + $C_2H_4^{2+}$ dissociation limit. In electron-molecule collisions, the ethane dication could be populated in different states, which favor different dissociation channels. Therefore

as shown in Supplementary Table 3 the present theoretical branching ratios extracted from the simulation for ground state do not agree well with the experimental ones.

Supplementary Table 3. Number of trajectories and branching ratios (BRs) of the two-body H_n^+ dissociation channels of $C_2H_6^{2+}$ dication.

Channel	Number of trajectories	Theoretical BR (%)	Experimental BR (%)
$H^+ + C_2H_5^+$	64	6.4	1.1
$H_2^+ + C_2H_4^+$ and $H_2 + C_2H_4^{2+}$	531	53.1	26.0*
$H_3^+ + C_2H_3^+$	230	23.0	72.9

* Only for the $H_2^+ + C_2H_4^+$ channel

Supplementary Note 3. Theoretical KER for different H_3^+ formation pathways

The simulated kinetic energy release (KER) is obtained as follows: (i) calculate the linear momentum and kinetic energy of every subunit, then (ii) sum their kinetic energies to produce the KER, and (iii) add up the remaining Coulomb potential energy of two charged units to obtain the final KER. The KER distributions for different H_3^+ formation pathways (i.e. transition state, and roaming mechanisms) are shown in Supplementary Figure 2.

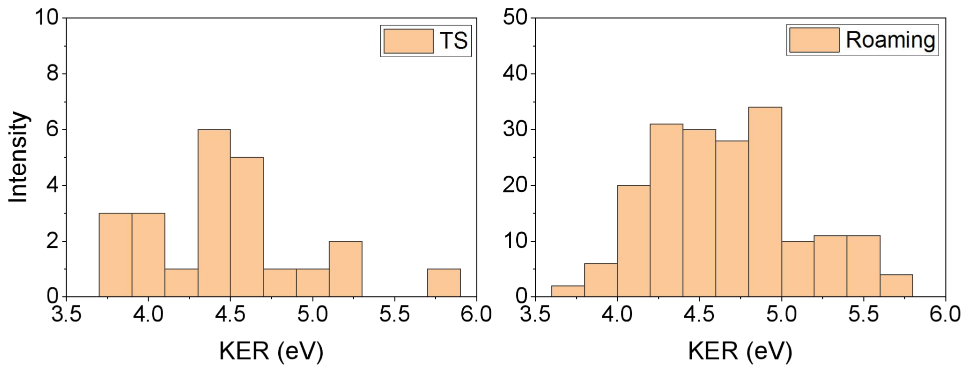

Supplementary Figure 2. KER distribution for different H_3^+ formation pathways.

Supplementary References

[1] K. Hoshina *et al*, J. Chem. Phys. **134**, 064324 (2011).

[2] P. Wang and C. R. Vidal, Chem. Phys. **280**, 309 (2002).

[3] T. Majima *et al*, Phys. Rev. A **90**, 062711 (2014).

[4] R. Kanya *et al*, J. Chem. Phys. **136**, 204309 (2012).

[5] Y. Zhang *et al*, Phys. Rev. A **100**, 052706 (2019).
