# Supplementary material for: Formation of H3+ from ethane dication induced by electron impact
Source: Commun Chem. 2020 Nov 9;3:160. doi: 10.1038/s42004-020-00415-9 (PMC9814254; doi:10.1038/s42004-020-00415-9)
Supplement: Supplementary file 2 — Description of Additional Supplementary Files [file 42004_2020_415_MOESM2_ESM.pdf]

Description of Additional Supplementary Files

File name: Supplementary Movie 1
Description: H_3^+ formation trajectory via transition state mechanism

File name: Supplementary Movie 2
Description: H_3^+ formation trajectory via H2 roaming mechanism

File name: Supplementary Movie 3
Description: H_3^+ formation trajectory via H roaming mechanism
